# Missouri Dental College

**Published:** 1882-05

**Authors:** 


### MISSOURI DENTAL COLLEGE.

The sixteenth annual commencement.

The number of matriculates for the session was sixteen.

The degree of D.D.S. was conferred on the following members of the graduating class by Professor H. H. Mudd, Dean of the Faculty:

| NAME. | REEIDENCE, | NAME. | RESIDENCE. |
|---|---|---|---|
| Chas. M. Bremermann | Missouri. | Thomas L. Gilmer | Illinois. |
| Ellis W. Bliss | Missouri. | Stephen D. McCallum | Canada, |
| George M. Cameron | Illinois. | Milton E. Taber | Minnesota. |

